# Tissue eosinophil level as a predictor of control, severity, and recurrence of Chronic Rhinosinusitis with Nasal Polyps

**DOI:** 10.3389/falgy.2025.1549332

**Published:** 2025-04-25

**Authors:** Julissa Vizcarra-Melgar, Serafín Sánchez-Gómez, Nuria López-González, Ramón Moreno-Luna, Jaime González-García, Juan Maza-Solano

**Affiliations:** ^1^ Department of Otorhinolaryngology and Head and Neck Surgery, Consorci Sanitari Integral, Sant Joan Despí Moisés Broggi Hospital, Barcelona, Spain; ^2^ Department of Surgery, University of Seville, Seville, Spain; ^3^ Department of Otorhinolaryngology and Head and Neck Surgery, Virgen Macarena Hospital, Rhinology Unit, Seville, Spain

**Keywords:** Chronic Rhinosinusitis with Nasal Polyps, eosinophilic inflammation, tissue eosinophilia, type 2 inflammation, disease control, biomarkers, recurrence

## Abstract

**Introduction:**

The histopathologic study of nasal polyps establishes endotype features of chronic rhinosinusitis (CRS). A tissular eosinophil count greater than 10 per high power field (HPF) classifies this condition as type 2 inflammation. Blood and mucosal eosinophils are suggested as biomarkers of severity and control of CRS. Additionally, a tissular eosinophil count greater than 55 per HPF has been related to a high risk of recurrence in the Asian population. Our study aims to determine whether tissue eosinophil count is associated with the control, severity, and recurrence of Chronic Rhinosinusitis with Nasal Polyps (CRSwNP).

**Methods:**

An observational study of patients with CRSwNP who underwent nasal mucosa biopsy was conducted between June 2021 and November 2023. Histopathologic features, asthma control, CRSwNP control and severity according to the POLINA consensus, quality of life parameters, recurrence of CRSwNP, and laboratory markers were recorded and compared with the tissular eosinophil count.

**Results:**

A total of 108 cases were included. The majority (70.4%) had concomitant asthma, with 31.5% of the cases having well-controlled disease. Most patients had uncontrolled (57.4%) and severe (62%) CRSwNP. Fifty-four cases underwent surgery and 43.5% experienced recurrence. More than half had a SNOT-22 score greater than 50 points. Eighty-one percent of patients had a tissular eosinophil count greater than 10 per HPF, and 60.2% had blood eosinophilia greater than $0.3\times 10^{3}$. Blood eosinophilia was related to CRSwNP severity and control. No significant differences were found between tissue eosinophil count and the severity, control, and recurrence of CRSwNP.

**Conclusion:**

Tissue eosinophil levels were not a marker of control, severity, and recurrence of CRSwNP in our data. Blood eosinophil levels, however, were a marker of CRSwNP control and severity.

## 1 Introduction

Chronic Rhinosinusitis with Nasal Polyps (CRSwNP) is a prevalent inflammatory condition affecting approximately 5%–12% of the global population, significantly impairing quality of life through persistent symptoms such as nasal obstruction, rhinorrhea, anosmia or hyposmia, and facial pressure. Its chronic nature and associated comorbidities, particularly asthma, which affects around 26%–48% of patients, highlight the need for comprehensive and effective management of both pathologies (1–3).

CRSwNP can be stratified into different endotypes based on the underlying immunological and inflammatory mechanisms. According to the EPOS 2020 guidelines, a tissular eosinophil count greater than 10 per high-power field (HPF) is a key criterion for defining the eosinophilic endotype of the disease (1, 3). This subtype is predominantly associated with type 2 inflammation, characterized by elevated IL-4, IL-5, and IL-13 cytokines (4, 5).

Eosinophil levels in peripheral blood and tissue have been suggested as disease severity, control, and recurrence biomarkers (6–15). Elevated tissue eosinophil counts have been linked to more severe symptoms, higher rates of polyp recurrence following surgical intervention, and poorer overall outcomes (7, 13, 15). Furthermore, regional variations have been observed. For instance, a study in an Asian population identified a tissue eosinophil count greater than 55 per HPF as a significant predictor of recurrence, emphasizing the heterogeneity of CRSwNP across ethnic groups (10). McHugh et al. further recommend this >55 eos/HPF cut-off as a reliable predictor of recurrence risk (12).

However, despite these findings, significant knowledge gaps remain regarding the prognostic value of eosinophil counts in specific populations, including Spanish patients. Most studies evaluating eosinophil-driven inflammation in CRSwNP have been conducted in Asian and North American cohorts, with limited data available from Southern European populations (12). Given that regional and ethnic differences in inflammatory patterns may influence disease progression and response to treatment, it is crucial to validate these findings in a Spanish cohort.

Considering the heterogeneous inflammatory profile of CRSwNP and its high recurrence rates following endoscopic sinus surgery (ESS), the identification of inflammatory biomarkers with prognostic value could play a crucial role in guiding the management and treatment of CRSwNP patients (11). This study aims to explore the correlation between tissue eosinophil counts and key clinical outcomes in CRSwNP, including disease control, severity, and recurrence.

## 2 Materials and methods

An observational retrospective cohort study was conducted at a tertiary referral hospital in Seville (Spain) from June 2021 to November 2023. Patients with CRSwNP who underwent nasal mucosa biopsy, either during consultation or surgery, were included; biopsy is routinely performed during the assessment of CRSwNP in our center. Patients younger than 18 years or those receiving topical or systemic corticosteroid therapy within 4 weeks before the biopsy were excluded.

CRSwNP was defined according to EPOS 2020 criteria, with bilateral diffuse disease, characterized by nasal polyps observed on endoscopy and diffuse inflammatory mucosal involvement in sinuses, confirmed by CT imaging (1). Disease control and severity were assessed based on the criteria outlined in the POLINA guidelines, a Spanish consensus on the management of CRSwNP (16). CRSwNP control was classified into three categories: controlled, partially controlled, and uncontrolled, based on the Visual Analogue Scale (VAS), the Sinonasal Outcome Test -22 (SNOT-22), the nasal polyps size on the endoscopy, the use of systemic corticosteroids in the last year and the need for surgery. Severity was categorized as mild, moderate, or severe based on VAS and/or SNOT-22 scores. Asthma control was evaluated using the Asthma Control Test (ACT) and classified as controlled, partially controlled and uncontrolled. All patients followed standard therapeutic guidelines of appropriate medical therapy, including intranasal corticosteroids (CIN) and saline flushes (SF) as first-line treatment (16).

Histopathological evaluation of tissue eosinophils was performed by a pathologist from our center with expertise in upper airway pathology. The eosinophil count was determined manually in hematoxylin-eosin (H&E) stained sections, selecting five high-power fields (HPF) at 400× magnification per sample. Tissue eosinophil counts were reviewed and categorized based on the number of eosinophils per HPF, classified as ≤10 per HPF, > 10 per HPF, or > 55 per HPF. Additional histopathological features, such as subepithelial edema and inflammatory cell predominance, were also analyzed. The pathologist was not blinded to clinical data, as tissue samples were processed according to standard pathology protocols.

All patients who underwent nasal mucosa biopsy during the study period and met the inclusion criteria were included in the study. The final sample size was determined by the total number of eligible patients who underwent biopsy between June 2021 and November 2023.

Patients who underwent surgery by two experienced sinus surgeons were followed up for over two years to assess post-surgical recurrence. The surgical procedures included limited functional ESS (L-FESS) or expansive FESS (E-FESS) as described in Martin et al. study (17). All patients were instructed to use intranasal corticosteroids and saline flushes after surgery according to the EPOS 4 patients guideline recommendations (18). Recurrence was defined by the presence of any of the following criteria: nasal polyps score ≥ 1, the requirement for oral corticosteroid therapy, the need for revision endoscopic sinus surgery, or a modified Lund-Kennedy mucosal edema score of 2 (12).

The study was approved by the Research Ethics Committee of Hospitales Universitarios Virgen Macarena-Virgen del Rocio (PIGE-0367-2019). Two authors (JVM, NLG) independently collected retrospectively data from medical records and carried out the assessment of disease control, severity and recurrence, while supervision was carried out by two additional authors (SSG and JML). Discrepancies in data collection were resolved by consensus among all authors. Evaluators were aware of the eosinophil values, as these were not criteria for classifying recurrence, severity, or disease control. Data collected included information on demographics and clinical characteristics, quality-of-life parameters (VAS, SNOT-22), endoscopic findings (Nasal Polyp Score (NPS), modified Lund Kennedy scale (LKM)), radiologic features (Lund Mackay (LM) scoring scale), laboratory markers and histopathologic characteristics.

Statistical analysis was conducted using IBM SPSS Statistics for Windows, version 26.0 (IBM Corp., Armonk, NY). Categorical variables were presented as frequencies and percentages, while continuous variables were expressed as mean ± standard deviation (SD) for normally distributed data or as median with interquartile range (IQR) for non-normally distributed data. The normality of variables was assessed using the Kolmogorov-Smirnov and Shapiro-Wilk tests. Comparisons between groups for continuous variables were performed using for independent samples *t*-test or ANOVA for normally distributed data and the Mann-Whitney *U* test or Kruskal Wallis for non-normally distributed data. Kaplan Meier disease-free survival curve was calculated and compared using the log-rank test or Breslow test.

## 3 Results

### 3.1 Patient demographics and clinical characteristics

The study included 108 patients, with a mean age of 54.9 ± 12.1 years. The gender distribution was 45.4% female and 54.6% male. Smoking was reported by 14.8%, and 24.1% had a known allergy to NSAIDs. Asthma was reported in 70.4% of the patients, and 40.7% had atopy (Table 1).

**Table 1:** Patient demographics and clinical characteristics.

Characteristics	N=108 n(%)
Age ($\bar{x}\pm SD$)	54.9±12.1
Gender	
Female	49(45.4)
Male	59(54.6)
Smoking	16(14.8)
NSAIDS allergy	26(24.1)
Asthma	76(70.4)
ACT ($\bar{x}\pm SD$)	19.1±5.2
Controlled	34(31.5)
Partially controlled	19(17.6)
Uncontrolled	16(14.8)
CRSwNP control	
Controlled	7(6.5)
Partially controlled	37(36.1)
Uncontrolled	63(57.4)
CRSwNP severity	
Mild	9(8.3)
Moderate	32(29.6)
Severe	67(62.0)
Atopy	44(40.7)
Prior sinus surgery	57(52.8)
ESS surgery	54(50.0)
Post-surgical recurrence (n=46)	20(43.5)
Recurrence time (months) ($\bar{x}\pm SD$)	8.7(6.7)

$\bar{x}\pm SD$, mean and standard deviation. NSAIDS, non-steroidal anti-inflammatory drugs; ESS, endoscopic sinus surgery.

Previous sinus surgery was performed in 52.8% of the patients. Half of the patients (50.1%) underwent ESS during the study period. Post-surgical recurrence was observed in 43.5%, with a mean recurrence time of 8.7 ± 6.7 months.

### 3.2 Endoscopic, radiological, and quality of life outcomes

The mean NPS for endoscopic evaluation was 4.82 ± 2.08, and the LKM scale showed a mean score of 6.7 ± 3.2. Radiological assessment using the Lund Mackay scale revealed a mean score of 12.5 ± 4.7.

Quality of life, measured using the SNOT-22 had a mean score of 57.4 ± 27.7, with 57.4% of patients scoring ≥ 50. The VAS for nasal symptoms showed a mean global score of 58 ± 31.5. Individual symptom scores for nasal obstruction, reduced sense of smell, nasal discharge, and facial pain were 66.3 ± 33.2, 82.2 ± 32.2, 65.0 ± 31.1, and 35.0 ± 35.6, respectively (Table 2).

**Table 2:** Distribution of endoscopic, radiological and quality of life outcomes.

Characteristics	N=108 n(%)
NPS (n=106) ($\bar{x}\pm SD$)	4.82±2.08
LKM scale (n=104) ($\bar{x}\pm SD$)	6.7±3.2
Lund Mackay scale (n=96) ($\bar{x}\pm SD$)	12.5±4.7
SNOT-22 (n=106) ($\bar{x}\pm SD$)	57.4±27.7
SNOT-22 ≥ 50 n(%)	62(57.4)
VAS (n=96) ($\bar{x}\pm SD$)	58±31.5
Nasal obstruction	66.3±33.2
Reduced sense of smell	82.2±32.2
Nasal discharge	65.0±31.1
Facial pain	35.0±35.6
Global	62.4±32.3

$\bar{x}\pm SD$, mean and standard deviation. NPS, nasal polyp score; LKM scale, modified lund kennedy scale; SNOT-22, sino-nasal outcome test -22; VAS, visual analogue scale.

### 3.3 Laboratory outcomes

Peripheral blood analysis showed a mean eosinophil count of 0.46 ± 0.39, with 60.2% of patients having eosinophil counts >300/mcL. The mean total IgE level of 597.2 ± 21.2 was recorded, with 40.7% having IgE levels >150 IU/mL. Additionally, 19.9% of patients presented IgE antibodies against Staphylococcus aureus (Table 3).

**Table 3:** Laboratory outcomes of included patients.

Characteristics	N=108
Total eosinophils in peripheral blood ($\bar{x}\pm SD$)	0.46±0.39
Eosinophils in peripheral blood > 300/mcL, n(%)	65(60.2)
Total IgE UI/mL ($\bar{x}\pm SD$)	597.2±21.2
IgE > 150 UI/mL, n(%)(n=94)	44(40.7)
IgE antibodies against Staphylococcus, n(%)(n=91)	18(19.9)

$\bar{x}\pm SD$, mean and standard deviation.

### 3.4 Histopathologic findings

Eosinophil counts ≤ 10 per HPF were observed in 18.5% of patients, while 81.5% had counts >10 per HPF, with 29.6% between 11–55 and 51.9% exceeding 55 per HPF.

Inflammation severity, assessed in 64 samples, was classified as mild in 20.2%, moderate in 22.2%, and severe in 39.8%. Regarding inflammatory predominance, eosinophilic inflammation was the most common (39.8%), followed by mixed (25.9%), lymphoplasmacytic (18.5%), neutrophilic (4.6%), and lymphocytic (3.7%).

Subepithelial edema was categorized as mild in 13%, moderate in 34.3%, and severe in 12%. Mucosal ulceration was observed in 14.2%, and dysplasia was rare, occurring in only 1.4% (Table 4).

**Table 4:** Histopathologic findings.

Characteristics	N=108 n(%)
Eosinophil count ≤ 10 per HPF	20(18.5)
Eosinophil count > 10 per HPF	88(81.5)
11–55 per HPF	32(29.6)
>55 per HPF	56(51.9)
Degree of inflammation (n=64)	
Mild	17(20.2)
Moderate	24(22.2)
Severe	43(39.8)
Inflammatory predominance (n=79)	
Lymphocytic	4(3.7)
Lymphoplasmacytic	20(18.5)
Eosinophilic	43(39.8)
Neutrophilic	5(4.6)
Mixed	28(25.9)
Subepithelial edema (n=64)	
Mild	14(13.0)
Moderate	37(34.3)
Severe	13(12.0)
Mucosa ulceration	15(14.2)
Dysplasia	2(1.4)

HPF, high power field.

### 3.5 Distribution of variables according to CRSwNP control

**Figure 1:**
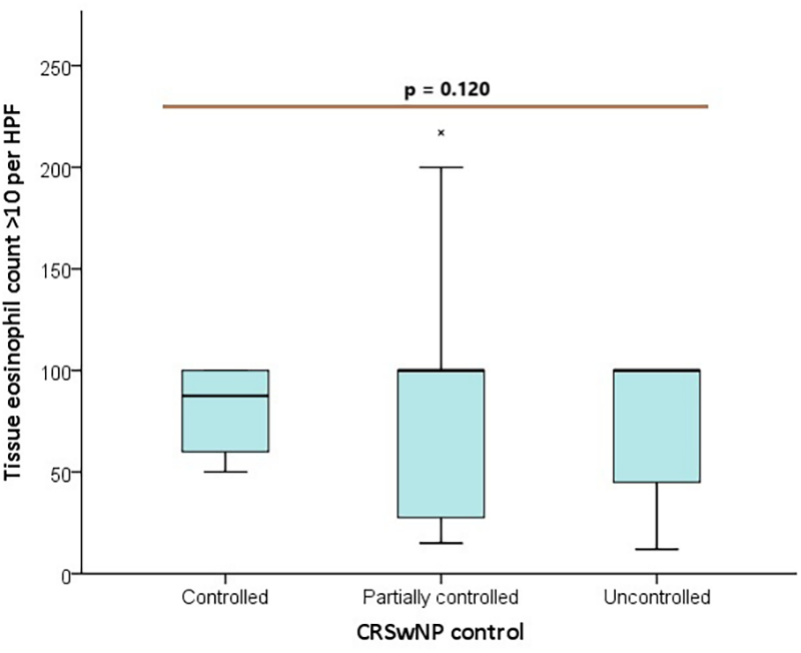
Box plot distribution of tissue eosinophil count > 10 per HPF between control groups.

**Figure 2:**
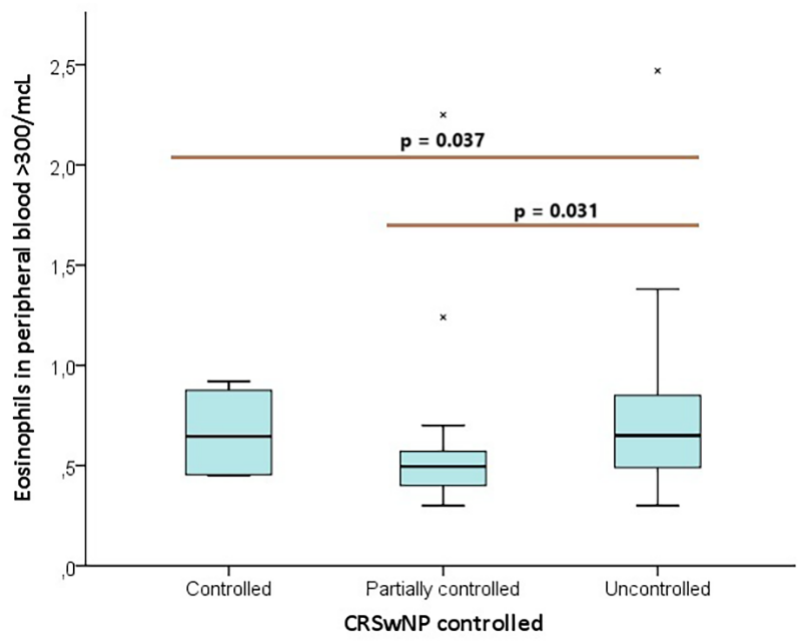
Box plot distribution of eosinophils in peripheral blood >300/mcL between control groups.

The analysis of eosinophil counts >10 per HPF among the controlled, partially controlled, and uncontrolled groups showed no significant differences (*p* = 0.120) (Figure 1). Specifically, eosinophil counts between 11–55 per HPF had a borderline *p*-value (*p* = 0.052), while counts >55 per HPF were not significantly different across the groups (*p* = 0.155). However, eosinophil levels in peripheral blood >300/mcL demonstrated a statistically significant difference between groups (*p* = 0.037), particularly between the uncontrolled and partially controlled groups (*p* = 0.031) (Figure 2, Table 5).

**Table 5:** Distribution of variables according to the CRSwPN control.

Variables	CRSwPN control (N=108)			*p*-value
	Controlled N=7	Partially controlled N=37	Uncontrolled N=63	
Eosinophil count > 10 per HPF Median (IQR)	87.5(42.5)	100(73.8)	100(57.5)	0.120^†^
11–55 per HPF Median (IQR)	50(0)	26(13.8)	40(25)	0.52^†^
> 55 per HPF Median (IQR)	100(32.5)	100(75)	100(0)	0.155^†^
Eosinophils in peripheral blood > 300/mcL Median (IQR)	0.64(0.4)	$0.49\;(0.2{)}^{*}$	$0.65\;(0.4{)}^{*}$	**0.037** ^†^

HPF, high power field; IQR, interquartile range.

^∗^Groups with statistical significance (p=0.031).

^†^*p*-value as determined by the Kruskall Wallis test.

Bold values indicate statistical significance (*p* < 0.05).

### 3.6 Distribution of variables according to CRSwNP severity

Eosinophil counts >10 per HPF did not significantly differ across mild, moderate, and severe CRSwNP severity groups (*p* = 0.709). The subgroup analysis for eosinophil counts of 11–55 per HPF (*p* = 0.150) and >55 per HPF (*p* = 0.630) also showed no significant differences. However, eosinophil levels in peripheral blood >300/mcL significantly differed among severity groups (*p* = 0.017), specifically between the uncontrolled and partially controlled groups (*p* = 0.016) (Table 6).

**Table 6:** Distribution of variables according to the CRSwPN severity.

Variables	CRSwPN severity (N=108)			*p*-value
	Mild N=9	Moderate N=32	Severe N=67	
Eosinophil count > 10 per HPF				
Median (IQR)	75(50)	75(75)	100(52)	0.709^†^
11–55 per HPF Median (IQR)	45(0)	25.5(17)	43.7(28)	0.150^†^
> 55 per HPF Median (IQR)	100(32.5)	100(0)	100(0)	0.630^†^
Eosinophils in peripheral blood > 300/mcL				
Median (IQR)	0.47(0.3)	$0.45\;(0.2{)}^{*}$	$0.61\;(0.4{)}^{*}$	**0.017** ^†^

HPF, high power field; IQR, interquartile range.

^∗^Groups with statistical significance (p=0.016).

^†^*p*-value as determined by the Kruskall Wallis test.

Bold values indicate statistical significance (*p* < 0.05).

### 3.7 Distribution of variables according to asthma control

Tissue osinophil counts >10 per HPF did not significantly differ across asthma control groups (*p* = 0.060). The analysis of other subgroups also showed no significant differences. Peripheral blood eosinophil levels >300/mcL were highest in the uncontrolled group but did not reach statistical significance (*p* = 0.456) (Table 7).

**Table 7:** Distribution of variables according to the asthma control.

Variables	Asthma control (N=69)			*p*-value
	Controlled N=34	Partially controlled N=19	Uncontrolled N=16	
Eosinophil count > 10 per HPF				
Median (IQR)	100(50)	50(67.5)	100(52.5)	0.060^†^
11–55 per HPF Median (IQR)	32(25)	25(25.5)	40(18.8)	0.259^†^
> 55 per HPF Median (IQR)	100(0)	100(25)	100(0)	0.659^†^
Eosinophils in peripheral blood > 300/mcL				
Median (IQR)	0.49(0.3)	0.58(0.4)	0.75(0.6)	0.456^†^

HPF, high power field; IQR, interquartile range.

^†^*p*-value as determined by the Kruskall Wallis test.

### 3.8 Post-surgery recurrence

A total of 54 patients underwent ESS, with 63.0% receiving L-FESS and 37.0% E-FESS. The comparison of patients with post-surgical recurrence and those without revealed no significant differences in age or gender distribution. A history of NSAID allergy was also comparable between groups, with 47.1% in the recurrence group and 52.9% in the non-recurrence group (*p* = 0.708). Comorbidities, including asthma and atopy, did not differ significantly between groups (Table 8).

**Table 8:** Post-surgery recurrence.

Characteristics	Recurrence		*p*-value
	Yes N=20	No N=26	
Age ($\bar{x}\pm SD$)	50±14.7	26.3±11	$0.100^{*}$
Gender			$0.588^{†}$
Female	9(42.9%)	12(57.1%)	
Male	11(44)	14(56%)	
NSAIDS allergy	8(47.1%)	9(52.9%)	$0.708^{†}$
Asthma	13(39.4%)	20(60.6%)	$0.373^{†}$
Atopy	3(27.3%)	8(72.7%)	$0.163^{†}$
SNOT-22 ($\bar{x}\pm SD$)	63.9±23.5	66.8±31.3	$0.704^{*}$
VAS score, Median (IQR)	77(26)	78(88)	$0.457^{‡}$
LKM ($\bar{x}\pm SD$)	7.8±2.7	6.2±3.5	$0.428^{*}$
Eosinophil count > 10 per HPF			
Median (IQR)	100(37.5)	100(50)	$0.892^{‡}$
11–55 per HPF ($\bar{x}\pm SD$)	40±14.14	35±13.41	$0.587^{*}$
> 55 per HPF Median (IQR)	100(0)	98(0)	$0.967^{‡}$
Eosinophils in peripheral blood > 300/mcL			
Median (IQR)	0.49(0.4)	0.50(0.4)	$0.900^{‡}$
IgE > 150 UI/mL Median (IQR)	1012.2(1584)	207.1(287)	$0.097^{‡}$

NSAIDS, non-steroidal anti-inflammatory drugs; ($\bar{x}\pm SD$), mean and standard deviation; IQR, interquartile range.

^∗^*p*-value as determined by the t-student test.

^†^*p*-value as determined by the chi-cuadrado test.

^‡^*p*-value as determined by the Mann-Whitney U.

Clinical outcome measures, including the SNOT-22 score, VAS score, and LKM, did not significantly differ between groups. The SNOT-22 score was 63.9 ± 23.5 in the recurrence group and 66.8 ± 31.3 in the non-recurrence group (*p* = 0.740). The VAS score median was 77 (IQR: 26) in the recurrence group and 78 (IQR: 88) in the non-recurrence group (*p* = 0.457). The median LKM score was 7.8 (IQR: 2.7) and 6.2 (IQR: 3.5) for recurrence and non-recurrence groups, respectively (*p* = 0.428).

**Figure 3:**
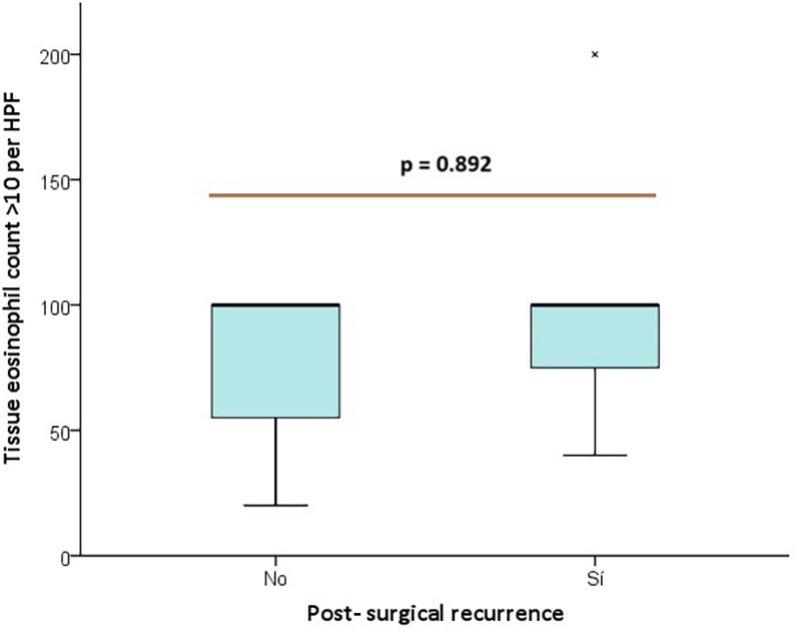
Box Plot distribution of tissue eosinophil count > 10 per HPF between post- surgical recurrence groups.

Histopathologic findings, including eosinophil counts >10 per HPF and eosinophil counts in peripheral blood >300/mcL, did not differ significantly between groups (Figure 3). Similarly, eosinophil counts in the 11–55 per HPF (*p* = 0.587) and >55 per HPF (*p* = 0.967) subcategories were not significantly different. Peripheral blood eosinophil counts >300/mcL and IgE levels >150 UI/mL were not considerably different between recurrence and non-recurrence groups.

**Figure 4:**
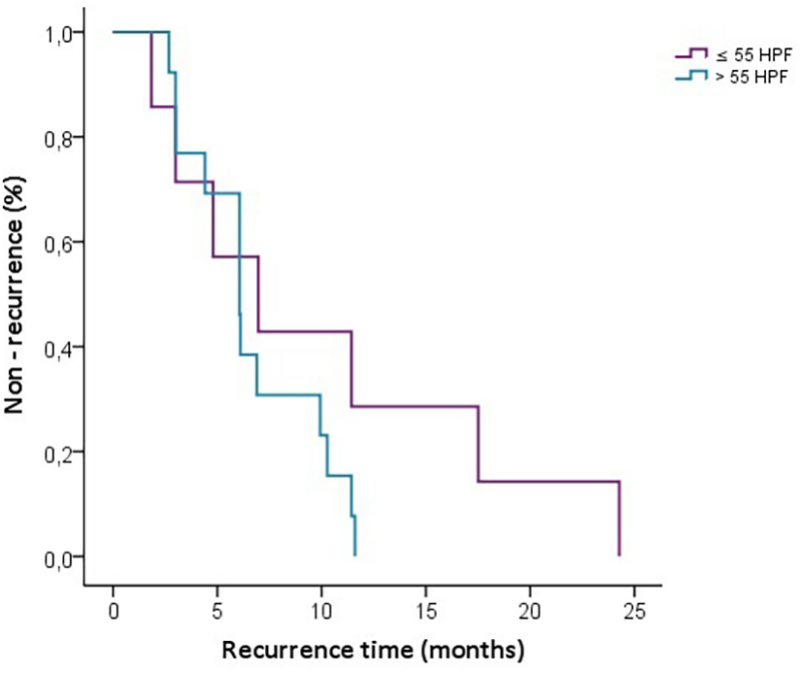
Kaplan-Meier curve for recurrence-free survival stratified by HPF Levels (≤55 vs. >55).

In the Kaplan-Meier graph, we observe the time to recurrence for patients with eosinophil cell counts >55 and ≤55, showing an earlier recurrence in those with values higher than 55. However, no significant differences were found between the groups when performing the Log-rank test (*p* = 0.216) (Figure 4).

## 4 Discussion

CRSwNP is a multifaceted inflammatory condition with significant clinical and therapeutic challenges (1). Despite advancements in medical and surgical management, recurrence remains a key obstacle, necessitating the identification of reliable predictors to optimize patient outcomes (1, 16). This study explored the association between clinical, laboratory, and histopathological variables and critical outcomes in CRSwNP, including disease control, severity, and recurrence. Blood eosinophil levels were significantly associated with disease control and severity, whereas tissue eosinophilia was not predictive of these outcomes or post-surgical recurrence.

This study represents one of the first applications of the POLINA guideline and its scales to assess control and severity (16). Previously, the only consensus on control was described in the EPOS and discussed on EPOS2020/EUFOREA expert opinion (1, 19). The POLINA guideline provides a structured framework for evaluating these parameters, incorporating the SNOT-22 score and the need for surgery criteria, offering a valuable alternative to the EPOS consensus, which previously served as the sole standard for defining disease control (16). By integrating the POLINA scales, this study explores their clinical applicability and highlights their potential to enhance the precision of CRSwNP assessment.

The concept of recurrence needs to be clearly defined in CRSwNP. Despite its clinical significance, there is no consensus on its definition. The EPOS 2020 defines this term as the return of a disease episode after a period without the problem and the recent EPOS 2020/EUFOREA expert opinion as the loss of remission that can occur either on- or off- treatment (1, 19). However, there is still a lack of specific clinical criteria to consistently identify and evaluate recurrence in routine practice. McHugh et al. meta-analysis described that for some authors, recurrence is strictly defined by the reappearance of nasal polyps visible on endoscopy within a specified postoperative timeframe, while others extend the definition to include any deterioration in symptom control, the requirement of oral corticosteroids or the need for revision surgery (12). This heterogeneity undermines efforts to establish standardized criteria for evaluating treatment outcomes in CRSwNP.

Eosinophilic inflammation has been widely recognized as a central feature of CRSwNP, particularly in the type 2 endotype. Previous studies have suggested that elevated eosinophil counts in both tissue and peripheral blood are associated with increased disease severity, poor control and a higher likelihood of recurrence. For instance, Soler et al. study describes detailed histopathologic findings, defending that the histological marker that showed a better correlation with severity was the eosinophil. They evaluated severity using CT, endoscopy findings, and smell tests (20). Another study by Aslan et al. in 2017 defends that having a tissue eosinophil count greater than 10 per HPF is related to disease severity and control (21). Furthermore, McHugh et al. research showed that tissue eosinophil was an acceptable marker for recurrence (12). Another most recent meta-analysis also defended that tissue eosinophilia and blood eosinophils are good markers of recurrence, the best biomarker was peripheral eosinophils (22). Our results align with these studies demonstrating high levels of eosinophils in our patients, indicating type 2 inflammation and therefore chronicity and severity of the disease. Also, in our series, statistical analysis found significant associations between eosinophils in peripheral blood and disease control and severity, but not with recurrence. We could not establish tissue eosinophilia as a control, severity, and recurrence predictor. This observation is consistent with the results of Gitomer et al., who also reported no significant relationship between tissue eosinophilia and disease severity (23). This may be attributed to the relative homogeneity of our cohort, given that most patients presented with severe CRSwNP, potentially minimizing differences in eosinophil levels across subgroups. Additionally, other contributing factors beyond eosinophils alone such as cytokines and chemokines were not evaluated (24, 25). Furthermore, factors such as the small sample size and the absence of longitudinal follow-up could have limited our capacity to identify significant associations.

Unlike a 2015 study conducted in China, which proposed a cutoff value of >55 eosinophils high per field as a predictor for disease recurrence, our findings did not replicate this association in our Spanish setting (10). This discrepancy may be attributed to differences in population characteristics, such as genetic, environmental, and healthcare-related factors, which vary between Asian and European settings. The Kaplan-Meier curve in our study provides an insightful visualization of recurrence times showing a trend toward earlier recurrence in patients with >55 eosinophils per high-power field compared to those with ≤55 eosinophils per HPF, although this observation lacked statistical significance. These findings suggest that while a threshold of 55 eosinophils may indicate potential recurrence risks, its predictive value appears less robust in our population.

Eosinophilic inflammation in CRSwNP has a significant clinical impact, particularly on loss of smell. According to the literature, the accumulation of tissue eosinophils contributes to functional impairment, as reflected in high loss on smell scores (26). In our study, we could confirm this, with the VAS for smell being the most affected. These findings are consistent with previous studies highlighting the relationship between eosinophilic inflammation and this key symptom (4, 22, 26, 27).

The role of other laboratory parameters, such as serum total IgE, remains controversial. Guo et al. highlight that, although serum total IgE is commonly elevated in patients with type 2 inflammation, its correlation with other biomarkers of type 2 inflammation, such as eosinophils and interleukins, is not always consistent. Moreover, their findings suggest that serum total IgE alone may not reliably reflect the severity or presence of mucosal inflammation in CRSwNP (28). These observations align with our findings, in which IgE levels failed to predict recurrence, disease control, or severity.

Eosinophilic CRSwNP results from a complex interplay between local and systemic type 2 inflammation. Locally, sinonasal inflammation is driven by epithelial dysfunction, eosinophil infiltration, and cytokines such as IL-4, IL-5, and IL-13. Systemically, IL-5 promotes eosinophil maturation in the bone marrow and their mobilization into circulation, sustaining chronic inflammation beyond the sinonasal mucosa (25). This supports the united airways hypothesis, linking CRSwNP with asthma and highlighting the need for targeted therapies addressing both local and systemic inflammation (16).

Asthma is a common comorbidity of CRSwNP, affecting 70% of the patients in our study. Interestingly, while the majority of these patients presented with controlled asthma, their CRSwNP remained uncontrolled. This paradox may be explained by the specificity of asthma treatments, such as inhaled corticosteroids and bronchodilators, which are primarily designed to target lower airway inflammation and may not effectively address the inflamed nasal mucosa characteristic of CRSwNP (29). In contrast to the findings of Wang et al., who identified asthma as a predictor of recurrence, our study did not establish a significant association between the presence of asthma and disease recurrence (30). This discrepancy could be attributed to differences in population characteristics and sample size between studies. Mukherjee et al. highlighted the correlation between elevated blood eosinophil levels and poor asthma control, as well as frequent exacerbations, emphasizing the complexity of interpreting systemic vs. localized markers of inflammation in predicting clinical outcomes (31). However, in our series, no significant association was observed between tissue eosinophil counts >10 per HPF and blood eosinophil levels, and asthma control. This could be attributed to the fact that the majority of participants had well-controlled asthma.

The analysis of other histopathological features revealed that the majority of patients (81.5%) had eosinophil counts >10 per HPF, reflecting the predominance of the eosinophilic endotype in CRSwNP (1). A significant proportion also exhibited subepithelial edema, consistent with the findings of Lee et al. 2021 study, which reported a high prevalence of this characteristic (32). Similarly, Cui et al. highlighted that subepithelial edema contributes to tissue remodeling and influences the clinical presentation of CRSwNP (33). Mucosal ulceration was observed in a few cases, as described in the Barham et al study (26). Furthermore, Shay et al. identified mucosal ulceration as a histopathologic parameter to consider, though they did not classify it as a hallmark of the disease (34). Only two cases of dysplasia were observed in our study, aligning with existing literature, which does not identify it as a common feature in CRS. This finding is consistent with the understanding that CRS is predominantly an inflammatory disease and is not typically associated with precancerous changes (32–34).

This study has some limitations, including its retrospective design and relatively small sample size, particularly in subgroup analyses, where disease control and severity were categorized into three subgroups each. Additionally, the cohort was relatively homogeneous, with most patients presenting severe CRSwNP, which may limit the generalizability of our findings. We also did not assess other inflammatory markers, such as cytokines or microbiota, which could further influence disease progression. Finally, treatment adherence was not systematically evaluated, which may have had some impact on disease control outcomes.

Ultimately, this study highlights the complex interplay of clinical, laboratory, and histopathological features in CRSwNP. From a clinical perspective, the application of the POLINA guideline offers a promising framework for standardizing disease evaluation. Additionally, blood eosinophil may serve as a useful tool for identifying patients at higher risk of poor outcomes and requiring closer monitoring or early escalation of therapy. Despite the limited predictive value of tissue biopsies in our study, local eosinophil count should be considered in the evaluation of CRSwNP, through more standardized sample collection requirements that ensure traceability of results (35). However, larger, multicenter studies with diverse populations and longitudinal follow-up are essential to validate these findings and refine predictive models for personalized management in CRSwNP.

## 5 Conclusion

Tissue eosinophil level was not a marker of control, severity, and recurrence of CRSwNP in our data. Blood eosinophil levels, however, were a marker of CRSwNP control and severity. Further research is needed to elucidate the mechanisms underlying the association between blood and tissue eosinophilia and the different phenotypes of this complex chronic disease, which could inform the development of more targeted therapies.

## Data Availability

The raw data supporting the conclusions of this article will be made available by the authors, without undue reservation.
